# CAPPRIC Study—Characterization of Community-Acquired Pneumonia in Spanish Adults Managed in Primary Care Settings

**DOI:** 10.3390/microorganisms9030508

**Published:** 2021-02-28

**Authors:** Jesús Molina, Amelia González-Gamarra, Leovigildo Ginel, Mª Encarnación Peláez, Juan Luis Juez, Antonio Artuñedo, Gonzalo Aldana, Enriqueta Quesada, Joan Josep Cabré, Antonio Gómez, Manuel Linares, Maria Teresa Marín, Pilar Yolanda Sanchez, Leonor Núñez, Jaime Gonzálvez, Enrique Mascarós, Javier López, Agustina Cano, José Herrero, María Carmen Serra, Enrique Cimas, Marta Pedrol, Juan Vicente Alfaro, Federico Martinón-Torres, Isabel Cifuentes, Cristina Méndez, Daniel Ocaña, on behalf of the CAPPRIC study group

**Affiliations:** 1 Primary Health Care Center Francia, Fuenlabrada, 28943 Madrid, Spain; 2 Primary Health Care Center Goya, 28009 Madrid, Spain; amelia.gamarra@gmail.com; 3 Primary Health Care Center Ciudad Jardín, 29014 Málaga, Spain; lginel@gmail.com; 4 Primary Health Care Center Puerta Blanca, 29004 Málaga, Spain; encarnitapg@hotmail.com; 5 Primary Health Care Center Urban, 48901 Barakaldo, Spain; juanluis.juezsenovilla@osakidetza.net; 6 Primary Health Care Center Nova Lloreda, 08917 Badalona, Spain; antoartugonza@gmail.com; 7 Primary Health Care Center Burriana II, 12530 Burriana, Spain; aldana_gon@gva.es; 8 Primary Health Care Service, Hospital de Alta Resolución “El Toyo”, 04131 Almería, Spain; quetiquesada@hotmail.com; 9 Primary Health Care Center Sant Pere, 43202 Reus, Spain; jcabre.tarte.ics@gencat.cat; 10 Primary Health Care Center Fernando el Católico, 50009 Zaragoza, Spain; agpeligros@gmail.com; 11 Primary Health Care Center Buenos Aires, 28038 Madrid, Spain; manuellinares@fundacionio.com; 12 Primary Health Care Center General Ricardos, 28019 Madrid, Spain; maite_marin@telefonica.net; 13 Primary Health Care Center Actur Sur, 50018 Zaragoza, Spain; ysanchez@comz.org; 14 Primary Health Care Center Barrio de la Salud, 38007 Santa Cruz de Tenerife, Spain; Leonuchi@gmail.com; 15 Primary Health Care Center Matamá, 36312 Vigo, Spain; gonzalvez.jaime@gmail.com; 16 Primary Health Care Center Fuente San Luis, 46013 Valencia, Spain; enmasba@gmail.com; 17 Primary Health Care Center Utrera Norte Príncipe de Asturias, 41710 Utrera, Spain; lopezantonioj95c@gmail.com; 18 Primary Health Care Center García Noblejas, 28037 Madrid, Spain; tinacano60@msn.com; 19 Primary Health Care Center Los Comuneros, 09006 Burgos, Spain; joseherreroroa@gmail.com; 20 Primary Health Care Center Just Ramírez, 46009 Valencia, Spain; maria.serrabartual39@gmail.com; 21 Primary Health Care Center Contrueces, 33210 Gijón, Spain; jcimash@gmail.com; 22 Primary Health Care Center Sant Llàtzer, 08221 Terrasa, Spain; MPedrol@CST.CAT; 23 Primary Health Care Center Murcia Centro-San Juan, 30003 Murcia, Spain; jvalfarog@hotmail.com; 24 Genetics, Vaccines and Pediatric Infectious Diseases Research Group (GENVIP), Healthcare Research Institute of Santiago de Compostela, 15706 Santiago de Compostela, Spain; federico.martinon.torres@sergas.es; 25 Medical Department, Pfizer S.L.U., Alcobendas, 28108 Madrid, Spain; cifuentesotero@pfizer.com (I.C.); Cristina.mendezdiez@pfizer.com (C.M.); 26 Primary Health Care Center Algeciras-Norte, 11205 Algeciras, Spain; ficusficus@gmail.com

**Keywords:** CAP, ambulatory, non-hospitalized, outpatient, pneumococcal CAP, community-acquired pneumonia

## Abstract

The real burden of community-acquired pneumonia (CAP) in non-hospitalized patients is largely unknown. This is a 3-year prospective, observational study of ambulatory CAP in adults, conducted in 24 Spanish primary care centers between 2016–2019. Sociodemographic and clinical variables of patients with radiographically confirmed CAP were collected. Pneumococcal etiology was assessed using the Binax Now^®^ test. Patients were followed up for 10 ± 3 days. A total of 456 CAP patients were included in the study. Mean age was 56.6 (±17.5) years, 53.5% were female, and 53.9% had ≥1 comorbidity. Average incidence of CAP was 1.2–3.5 cases per 1000 persons per year. Eighteen patients (3.9%) were classified as pneumococcal CAP. Cough was present in 88.1% of patients at diagnosis and fever in 70.8%. Increased pulmonary density (63.3%) and alveolar infiltrates with air bronchogram (16.6%) were the most common radiographic findings. After 14.6 ± 6.0 days (95% CI = 13.9–15.3), 65.4% of patients had recovered. Hospitalization rate was 2.8%. The most frequently prescribed antibiotics were quinolones (58.7%) and β-lactams (31.1%). In conclusion, one-third of CAP patients did not fully recover after two weeks of empiric antibiotic therapy and 2.8% required hospitalization, highlighting the significant burden associated with non-hospitalized CAP in Spain.

## 1. Introduction

Lower respiratory infections have been a leading global infectious cause of mortality in all age groups over the past twenty-five years [1]. Among them, community-acquired pneumonia (CAP) is a major cause of morbidity and mortality worldwide, especially in the elderly. It is responsible for approximately 1 million hospital admissions yearly, producing a considerable impact on health care resources. The incidence of CAP is highly variable and differs depending on the country, study population, setting and study approach. In the USA, for instance, the reported incidence of CAP requiring hospitalization between 2014–2016 was 6.5 patients per 1000 adults [2], while in Europe, the incidence of CAP ranged from 7.6 to 14 cases per 1000 adults in patients older than 65 years [3]. In Spain specifically, the incidence of CAP in primary care patients ≥ 65 years was 24.8–94.8 per 1000 persons [4]. However, most of these studies are based only on hospital data, although the majority of CAP patients receive outpatient treatment [5].

The diagnosis of CAP is primarily based on clinical manifestations and signs of lower respiratory tract infections. Suspected CAP should be confirmed by chest radiography and microbiological testing. However, despite various diagnostic tests currently available for CAP, its etiology is only identified in around 50% of cases [6]. Among the different possible causative CAP agents, the bacteria *Streptococcus pneumoniae* (*S. pneumoniae*) is the most frequently identified pathogen [7]. In fact, it was found to be the leading cause of lower respiratory infection morbimortality at a global level during 1990–2016 [8]. Its prevalence in CAP is usually underestimated due to a lack of sensitive and specific tests. A traditional diagnostic approach for CAP includes blood or sputum culture [9], but although cultures are very specific to confirm the etiology of the disease, they are not routinely used in primary care facilities. Alternatively, non-invasive rapid tests like Binax Now^®^ allow the specific identification of *S. pneumoniae* antigen in CAP patients by detecting urinary C-polysaccharide antigen, a component of the pneumococcal cell wall that is excreted in the urine [10].

Most studies on CAP have been based on hospitalized patients, yet most patients with CAP are treated on an outpatient basis. Only a few epidemiological studies have focused on the contribution of CAP treated in an ambulatory setting in Spain [4,11], and these are restricted to specific regions or age groups. The last Spanish epidemiologic study on CAP in primary care was performed between 2009 and 2013 [4], reinforcing the need for up-to-date data on this specific population.

Despite the recognized importance of ambulatory CAP in adults, there are scant data in the literature regarding the burden of the disease in this setting. This prospective study aimed to analyze the clinical characteristics, management, and evolution of radiologically confirmed CAP in adults seen in primary care centers in Spain during a three-year period (2016–2019). 

## 2. Materials and Methods 

### 2.1. Study Design

This prospective, multicenter, population-based active surveillance study was designed to evaluate the incidence and characteristics of ambulatory CAP confirmed by chest radiography in adults treated in 24 primary care centers between April 2016 and April 2019. 

The study was conducted in accordance with the Declaration of Helsinki, and the protocol was reviewed and approved by the Institutional Ethics Committee of Madrid (Spain) (reference PFI-PRE-2015-01) and at all participating centers, according to local standards. Informed consent was obtained from all patients. The study was non-interventional, and patients were managed according to the criteria of their treating physicians. 

### 2.2. Patients

Eligible participants were adults 18 years of age or older who visited participating primary care physicians at study sites and had signs and symptoms of pneumonia, including two or more of the following: fever (temperature >38 °C) within 24 hours prior to recruitment, hypothermia (<35.5 °C) measured by a healthcare professional within 24 hours prior to recruitment, shaking chills, pleuritic chest pain, cough, expectoration, dyspnea, tachypnea, general discomfort, auscultation abnormalities indicative of pneumonia (crackles) or indications of lung consolidation (dullness on percussion, bronchial breath sounds, or egophony). 

Patients who required hospitalization at study inclusion in the opinion of the physician were excluded from the study. Other exclusion criteria included: the presence of CAP within the 60 days prior to the screening visit; hospital inpatient; diagnosis of active pulmonary tuberculosis according to the medical history; post-obstructive pneumonia due to lung cancer, vaccination with a pneumococcal vaccine in the 10 days prior to the screening visit; previous recruitment in this study in the 60 days prior to the screening visit; and pregnant or potentially pregnant women at screening visit.

After giving their written informed consent, patients underwent the following selection procedures to be finally included in the study: (i) Collection of a urine sample for pneumococcal antigen testing (Binax Now^®^); and (ii) Chest x-ray performed according to standard clinical practice within 72 hours before or after selection for the study. 

The Binax Now^®^ test was performed according to the manufacturer’s instructions [12].

### 2.3. Outcomes

The main study objective was to analyze the clinical characteristics, management and evolution of CAP confirmed by chest radiography in adults 18 years of age and older in ambulatory care in Spain. We also aimed to specifically identify the proportion of pneumococcal CAP using the Binax Now^®^ test in urine.

Secondary objectives included description of the demographic characteristics, underlying diseases, and risk factors in CAP patients ≥ 18 years. We also estimated the incidence rate of ambulatory radiographically confirmed CAP in those centers where a population denominator was established.

The incidence rate of outpatient CAP was calculated in the four participating centers (Burriana II, Urban, Buenos Aires and Ciudad Jardín) where a population denominator could be established. These primary care centers belonged to different geographical regions in Spain (Valencian Community, Basque Country, Community of Madrid, and Andalusia). All patients ≥ 18 years with CAP confirmed by chest X-ray during the study recruitment period were included.

### 2.4. Statistical Analysis

In order to determine the percentage of pneumococcal CAP confirmed both by radiography and the Binax Now^®^ assay, a descriptive analysis of all the variables was performed. Study estimates were made with a 95% confidence interval (significance was considered as a *p*-value < 0.05). All calculations were done using the SAS Enterprise Guide 7.1 statistical package (SAS Institute Inc., Cary, NC, USA). 

## 3. Results

### 3.1. Patient Characteristics

Among 851 adult patients with CAP registered between April 2016 and April 2019 in 24 Spanish primary care centers, 524 patients were considered for inclusion in the study. Of these, 68 (12.5%) were ineligible because they did not meet the inclusion criteria. A total of 456 (84.1%) patients were finally enrolled and subsequently analyzed. Twenty-eight patients were lost to follow-up and five were hospitalized at the time of the follow-up visit. Finally, 422 patients attended a follow-up visit 10 ± 3 days after the screening visit. The average incidence rate of CAP calculated in four of the participating centers varied from 1.2 to 3.5 cases per 1000 persons per year (Table 1). Registered cases of CAP were more frequent in the winter months, especially in January and February.

Eighteen (3.9%) of the cases were classified as pneumococcal CAP and the rest (438) as non-pneumococcal CAP according to the results of the Binax Now^®^ test. The mean age of the participants was 56.6 years (±17.5), and 53.5% were female. Patients from 17 different nationalities participated in the study, most of them (93.93%) Spanish.

### 3.2. Clinical Signs and Symptoms

Cough, fever and expectoration were the most common symptoms of pneumonia detected at recruitment in 88.16%, 70.83% and 53.73% of the patients, respectively (Table 2). Expectoration was significantly more present in patients with pneumococcal CAP than in patients with non-pneumococcal CAP (77.78% vs. 52.74%, *p* = 0.038). The frequency of patients with fever and cough was also significantly higher in the sub-population of patients with no underlying conditions than in those with one or more underlying conditions (79.05% vs. 63.82% for fever, *p* = 0.0004; 91.43% vs. 85.37% for cough, *p* = 0.046). Conversely, dyspnea was more common in patients with ≥1 underlying condition (38.21%) than in patients with no underlying conditions (24.76%).

Regarding radiographical findings consistent with CAP, we found that 372 patients (81.58%) had increased lung density due to infection and 141 patients (30.92%) had alveolar infiltrates with air bronchogram.

### 3.3. Underlying Conditions

The clinical history of the study population is summarized in Table 3. Just over half of the patients (53.95%) presented one or more underlying conditions. The most frequent underlying conditions were diabetes, asthma and chronic obstructive pulmonary disease (COPD), reported in 11.84%, 8.55% and 7.46% of affected patients, respectively. Nearly 15% of cases among CAP patients were active smokers or ex-smokers for less than 6 months. We found no significant differences between patients with confirmed pneumococcal CAP and non-pneumococcal CAP. Previous pneumococcal vaccination was reported in 83 patients (18.20%); of these 67 (80.72%) had received the polysaccharide vaccine and 27 (32.53%) the PCV13 vaccine. One hundred and sixty-nine CAP patients (37.6%) had received the influenza vaccine. Previous history of pneumonia and influenza vaccination was significantly lower in patients with no underlying conditions (6.19 vs. 28.46% for pneumococcal vaccine and 20.95% vs. 50.81% for influenza vaccine, *p* < 0.0001).

### 3.4. Antibiotic Treatments

Almost all the patients studied (*n* = 455) received antibiotic treatment with an average treatment duration of 9.9 ± 2.7 days (95% CI = 9.72–10.22). Quinolones and β-lactams were the most common antibiotics prescribed, used in 58.77% (*n* = 268) and 31.14% (*n* = 142) of patients, respectively (Table 4).

### 3.5. Outcome

A total of 422 patients attended their follow-up visit at 10 ± 3 days after the screening visit. The percentage of clinical recovery was 65.40% and mean time to recovery was 14.64 ± 6.04 days (95% CI = 13.93−15.36). Eight patients were hospitalized within the follow-up period. Mean length of hospital stay was 5.63 days (95% CI 3.79–7.46). Globally, the hospitalization rate after inclusion in the study was 2.8% (*n* = 13), and no deaths were reported.

## 4. Discussion

Our data provide an updated overview of the characteristics of radiologically confirmed CAP in adults managed in primary care in Spain. We were also able to obtain a current picture of ambulatory CAP patients by describing their clinical and demographic characteristics in addition to the treatments they received, showing that one in three patients does not recover within 7–10 days after starting antibiotic therapy. 

Previous studies have examined the significance of ambulatory CAP in the Spanish adult population. A prospective epidemiological study that analyzed the incidence of both hospitalized and ambulatory CAP in adults in the north-east region of Spain during 2002–2005 reported an annual incidence of 2.6 cases per 1000 elderly persons [13], while a study by Aramburu et al. found an incidence rate of 8.3 cases per 1000 inhabitants and year in the population > 14 years in the north of Spain (Gipuzkoa) [11]. Our results are based on the incidence data of four of the participating primary care centers with average rates ranging from 1.2 to 3.5 cases per 1000 persons per year, with a mean of 2.1 cases per 1000 persons per year. The variability in our data might be explained by differences in the procedure for recording CAP cases in each of the primary care centers included. Thus, our mean incidence could be an underestimation of the real burden of CAP managed on an outpatient basis in Spain. In fact, a slightly higher incidence was reported in another nationwide study performed between 2009 and 2013 in adults with CAP from primary care, with an incidence of 4.63 per 1000 persons/year [14]. In contrast, the discrepancies observed between previous studies and ours could be attributed to differences in the population studied. While some of the studies focused on CAP in patients older than 65 years or included the population between 14 and 18 years, our data were based on adults aged 18 years and older. The region included also differs between these three studies. 

*S. pneumoniae* is globally the leading etiological cause of low-respiratory infections causing more than five times more years lived with disability than the second leading cause (influenza) [15]. In fact, it is also the most frequently identified CAP causative pathogen in ambulatory care, with an estimated prevalence of around 35% [16]. However, among the 456 patients analyzed in our study, only 18 (3.9%) were positive for *S. pneumoniae* antigens. One possible explanation for the under detection of pneumococcal CAP in our sample is the low sensitivity of the urine detection test in ambulatory patients. In fact, previous studies have already reported modest sensitivity of the Binax Now^®^ test in adult patients with moderate pneumonia with respect to those classified as severe pneumonia [17]. Interestingly, a Spanish prospective, laboratory-based study also found that the sensitivity of the pneumococcal urinary antigen tests fell from 77.9%, in the period from 2006 to 2010, to 60.5% between 2011 and 2015 [18]. These variations were associated with changes in C-polysaccharide composition depending on the serotype, producing variations in the sensitivity of urine antigen tests that ranged from 33.3% to 100% depending on the serotype [18]. Moreover, we found that 30.9% of patients presented alveolar condensation with air bronchogram, a radiographic finding frequently associated with pneumococcal pneumonia, reinforcing the hypothesis of underestimation of CAP cases by the Binax Now^®^ test in our sample.

About half of our study population presented one or more comorbidities, being diabetes, asthma and COPD the most commonly reported. Chronic respiratory diseases, including asthma and COPD, were the third leading cause of death in 2017, with an important impact in the quality of life of the patients [19,20]. Intriguingly, substantially increased rates of pneumococcal disease have been observed among persons with underlaying risk conditions, such as diabetes or asthma [21]. On the other hand, it is important to consider the negative impact of CAP and pneumococcal disease in the evolution, quality of life and survival of patients with chronic diseases [22]. Therefore, it is crucial to take into consideration the presence of underlying conditions in order to understand the real burden of the disease and, consequently, appropriately target the interventions to reduce the risk of infection.

In our cohort, 46.0% of patients with clinically confirmed CAP did not present any underlying condition. It is interesting that almost half of the radiographically confirmed CAP patients did not have any underlying factors related with CAP, such as COPD or smoking history. Indeed, the study performed by Rivero-Calle et al. in Spanish adults with CAP in primary care between 2009–2013 showed that the incidence of CAP was strongly associated with a high prevalence of multiple lifestyle risk factors and comorbidities [4]. Specifically, less than 30% of the adult CAP patients included in the study showed no risk factors, while patients >55 years had an identified risk factor in 85.7% of cases [4]. Nevertheless, our study did not specifically analyze the association between risk factors and presence of CAP, as in the aforementioned study. Furthermore, the fact that 65.4% of our patients recovered from CAP in around 10 days suggests that a significant percentage of the population included in the study presented a mild form of CAP, more likely not associated with an underlying condition. 

We found that dyspnea was significantly more common among patients with one or more underlying conditions, than in patients with no clinical history of interest. Since increased perception of dyspnea has been observed in patients with respiratory diseases and in persons with high anxiety/depression levels [23], one could argue that the higher number of patients with dyspnea in our study could, in fact, be associated with the presence of other comorbidities. Previous vaccination with both influenza and pneumonia vaccines was also significantly more frequent in the sub-population of patients with underlying conditions, suggesting that, as recommended by the national health authorities [24,25], people with comorbidities are receiving vaccines against flu and pneumonia, albeit with a low uptake, especially for the pneumococcal vaccine.

Multidisciplinary guidelines for the management of CAP (GNAC) together with the guidelines of the Spanish Society of Pulmonology and Thoracic Surgery (SEPAR) are used by the majority of healthcare professionals in Spain who participate in the care of CAP patients [16,26,27]. In terms of the treatment of ambulatory CAP, they recommend a 5–7 day course of quinolones (levofloxacin and moxifloxacin) or dual antibiotic therapy of amoxicillin and a macrolide [16]. This is consistent with the results found in our cohort, with quinolones and β-lactams being the most common antibiotics prescribed (58.77% and 31.14% of patients, respectively), demonstrating that primary care physicians in Spain are correctly following national guidelines. However, we found that the mean duration of treatment was almost 10 days, suggesting that even for moderate CAP cases that do not require hospitalization, professionals are prescribing antibiotic cycles that exceed the necessary duration, even when it has been demonstrated that shorter cycles are more effective in the treatment of CAP [28,29]. 

This study has some limitations. First, the incidence rate for ambulatory CAP was only calculated in four of the participating centers, and it provides therefore an estimation of the real incidence of non-hospitalized CAP in Spain. Second, the number of confirmed cases of pneumococcal CAP was too low, preventing us from making predictions and association analysis. We may also have missed some CAP cases that were not diagnosed initially in primary care. Nevertheless, our study also has some important strengths. It is a prospective study that includes 24 primary care centers throughout Spain, thus providing a global image of what is happening nationwide. Furthermore, it is important to highlight its population-based design, and that all cases of CAP were radiologically confirmed.

In summary, this multicenter prospective study offers insights into the current situation of non-hospitalized CAP patients aged ≥18 years in Spain, and provides an updated picture of their clinical characteristics, management and evolution, thus increasing awareness of the current burden of the disease treated at primary care level. 

## Figures and Tables

**Table 1:** Estimated incidence rates of community-acquired pneumonia (CAP) in four of the participating primary care centers.

Primary Health Care Center (Spanish Region)	Cases	Population	Overall Incidence Rate Per 1000 Persons (95% CI)	Average Incidence Rate ^1^ Per 1000 Persons Per Year
Ciudad Jardín (Andalusia)	216	29,144	7.41 (6.51–8.53)	2.47
Urban (Basque country)	58	8767	6.62 (5.12–8.54)	2.21
Buenos aires (Community of Madrid)	76	20,874	3.64 (2.91–4.55)	1.21
Burriana II (Valencian Community)	88	8369	10.51 (8.54–12.94)	3.50
	**Total**	67,154	6.52 (5.94–7.16)	2.18

^1^ Incidence rate calculated in all patients ≥18 years with CAP confirmed by chest X-ray during the study recruitment period from the four participating centers where a population denominator was established.

**Table 2:** Clinical presentation of study population in the whole cohort.

		Total Cohort (*n* = 456)
Signs and symptoms * **n (%)**	Fever	323 (70.83%)
	Chills	187 (41.01%)
	Cough	402 (88.16%)
	Expectoration	245 (53.73%)
	Pleuritic chest pain	166 (36.40%)
	Dyspnea	146 (32.02%)
**Radiographic features ^#^** **n (%)**	Pleural effusion	18 (3.95%)
	Increased lung density due to the infection	372 (81.58%)
	Alveolar infiltrates with air bronchogram	141 (30.92%)
	Multilobar	9 (1.97%)
	Lobar	89 (19.52%)
	Segmental	43 (9.43%)
	Other radiographic findings	15 (3.29%)

* All patients presented at least two of the signs and symptoms listed. Fever was defined as temperature higher than 38 °C in the 24 hours prior to recruitment. ^#^ Radiographic findings were not exclusive.

**Table 3:** Underlying conditions in the study population.

		Total Cohort (*n* = 456)
**Underlying conditions** **n (%)**	No	210 (46.05%)
	Yes	246 (53.95%)
	COPD	34 (13.82%)
	Asthma	39 (15.85%)
	Congestive heart failure	15 (3.29%)
	Coronary disease	24 (9.76%)
	Chronic kidney disease	15 (3.29%)
	Cerebrovascular disease	10 (4.07%)
	Diabetes	54 (21.95%)
	Previous pneumonia ^§^	13 (2.85%)
	Smoker *	66 (14.47%)
	Alcoholism ^#^	3 (0.66%)
**Vaccination history** **n (%)**	Previous pneumococcal vaccine	83 (18.20%)
	PPSV23	67 (80.72%)
	PCV13	27 (32.53%)
	Influenza	169 (37.06%)

Percentages calculated over the sample with 1 or more underlying conditions. ^§^ Previous pneumonia within the last 6 months. * More than 10 cigarettes/day (0.5 pack-years) in the last year or ex-smoker <6 months. ^#^ Alcohol intake ≥80 g/day for at least the previous year.

**Table 4:** Antibiotic treatments used in CAP managed in primary care.

		Total Cohort (*n* = 456)
β-lactam		142 (31.14%)
Cephalosporins		21 (4.79%)
Macrolides		19 (4.17%)
Quinolones		268 (58.77%)
Others		6 (1.32%)
Antibiotic combinations	Total	23 (5.04%)
	Macrolide + β-lactam	9 (39.13%)
	Quinolone + β-lactam	5 (21.74%)
	Other antibiotics combination	9 (39.13%)

## Data Availability

No new data were created or analyzed in this study. Data sharing is not applicable to this article.
